# Painful Menstruation and Its Treatment

**Published:** 1894-03-03

**Authors:** James Oliver

**Affiliations:** Physician to the Hospital for Women, London


### PAINFUL MENSTRUATION AND ITS TREATMENT.

By James Oliver, M.D., F.L.S., F.R.S.(Edin.), Physician to the Hospital for Women, London.

In this and other civilised countries of the world pain is frequently complained of in association with that increased determination of blood to the genital organs which takes place correlatively with menstruation. In the case of the women of savage peoples it is difficult to obtain reliable information regarding the evolution of this physiological phenomenon. After careful inquiry, I am inclined to believe that so long as these women adhere to their primitive mode of life this functional variation recurs without the individual being conscious of its occurrence, except, of course, in so far as its external manifestation is concerned. If this opinion be generally maintained then the physico-chemical variations, which produce pain during menstruation, are dependent upon changes in our mode of life which have been engendered by civilisation.

Under ordinary circumstances we are not conscious of physiological variations taking place in the organs of our bodies, yet the function of menstruation is seldom performed without a certain amount of correlative discomfort or pain being experienced. The organs of generation, it is well known, are highly impressionable, and this, no doubt, may account for the fact that these organs above all others should reveal the prejudicial influences of civilisation.

In many cases the pain complained of in association with menstruation is due to some decided organic change in or around the organs of generation. It may be that the ovaries and tubes have become more or less involved in adhesions consequent upon an attack of pelvic peritonitis, or that some neoplasm, such as a fibroid, has begun to develop in the uterus, and to one or other of these or some similar causes the pain may be attributable.

It is not, however, my intention to deal at present with this, the organic class of case, but with that obscure group in which pain of greater or less severity is experienced during, and has practically recurred ever since the establishment of menstruation, and in which a most careful examination of the pelvic organs fails to detect in or around them any evidence of change of a pathological character. The organs concerned in the process of reproduction appear, in fact, to be structurally healthy.

The phenomena of pain are among the most obscure and intricate that we are called upon to deal with, and at no time perhaps do we appreciate the truth of this dogma so much as when we come to investigate the cause or causes of those changes which, resulting in association with menstruation, produce pain. The difficulty is manifestly increased, too, by the fact that we have to deal with changes in a nervous system the sympathetic—whose functions are imperfectly known, and whose structure is such that, in consequence of the absence of insulating material, disturbances originating in one fibre may be propagated by simple contiguity to neighbouring fibres. For this reason the pain of menstruation is invariably more or less diffuse, although referred to the lower abdomen.

I have seen cases in which the severe pain of menstruation was held in abeyance, whilst this function was performed regularly during lactation. Immunity from pain under such circumstances is probably accounted for by the fact that the overplus of energy, which would have induced pain, is directed to and spent upon the mammary glands.

From clinical observations, I am disposed to divide the cases of functional dysmenorrhœa into four classes.

*Class* 1.—Those in which the pain is due to some physico-chemical variation in the nervous apparatus.

Menstruation, as I have elsewhere maintained, is determined by a spontaneous evolution of nerve energy by a liberation of motion, which results from chemical changes taking place in certain nerve vesicles located in the medulla oblongata. Regarding those states of nerve tissue which yield pain it is true we know absolutely nothing; nevertheless, it is evident that the pain experienced during menstruation, if the nerve origin of this manifestation be accepted, may be attributable to some abnormal variation in the physico-chemical state of the nervous apparatus which participates in the evolution of this phenomenon. The pain complained of in association with menstruation is most commonly referred to the lower abdomen; it does not, however, follow that the cause of the pain is located at the periphery—in the ultimate filaments—it may be located in the nerve trunk, or in the sensori-motor centre as well. If the disturbance is located in the sensori-motor centre the pain may be referred to the head or to the abdomen, but it rarely happens that it is referred to both regions at the same time, or even during any given menstrual epoch.

*Class* 2.—Those in which the pain is due to some want of harmony on the part of the blood vessels which should respond to the changes in the nerve centre.

Prior to and during menstruation there is an increased determination of blood to the genital tract. It is quite possible, however, that the blood vessels may not uniformly respond to the call made upon them, and here and there instead of dilating vessels may become more or less contracted. The pain of menstruation may therefore be due to the blood being forcibly driven against a vessel, or a portion of a vessel, which is unduly constricted. This vaso-motor spasm is usually, however, of short duration, and yields, as a rule, readily to the application of heat.

*Class* 3.—The pain may be due to some physico-chemical disturbance in the excreting structures.

The menstrual discharge is excreted by glands which are located more especially in the uterus, and under ordinary circumstances this discharge is fluid. In many of those cases, however, in which pain is experienced during menstruation the excretion is more or less desiccated and lumpy, and although at present we are unable to attribute this alteration in character to a definite cause, it is, nevertheless, evident that the chemical composition of the fluid is deranged.

*Class* 4.—The pain may be caused partially or entirely by some disturbance of the gut.

The lower portion of the large bowel is not infrequently disturbed during menstruation, as diarrhœa is often observed either before or during the evolution of this phenomenon. And it is more than probable that in many of the cases of dysmenorrhœa the pain is due to spasm in some portion of the gut, or to fermentative changes set up in the contents of this structure.

*Treatment.*—To our social life I attribute those changes which are the cause of pain during menstruation. If, therefore, this scourge is to be mitigated or abolished, we must regulate carefully the food, clothing, and mode of life generally of the girl. The functions of the skin and kidneys should be as well maintained as possible, and constipation should be guarded against. If the pain is due to some physico-chemical disturbance in the nervous apparatus, phenazonum in five grain doses every half hour or hour will prove beneficial, and it should be repeated if necessary until six doses are taken. This drug, when administered to the extent of thirty grains, tends to lessen the menstrual discharge. If, therefore, this excretion is in any given case of dysmenorrhœa invariably scanty, a large dose of quinine should be administered occasionally at night during the intermenstrual period, as otherwise the phenazonum may prove inefficacious. If the pain is due to vasomotor spasm and is relieved by warmth, then liquor æthyl nitritis, amyl nitrite, or nitroglycerine will be found most advantageous. If the excretion is unusually thick and altered in colour, chloride of ammonium or some lime salt, such as chloride of calcium or hypophosphite of lime should be administered during the intermenstrual period, and five-grain doses of phenazonum during the paroxysm. If the pain is due to some disturbance of the gut, some preparation of ammonia or chinoline or grindelia should be administered.

